# A commentary on the 2015 Canadian Clinical Practice Guidelines in glutamine supplementation to parenteral nutrition

**DOI:** 10.1186/s13054-015-1175-3

**Published:** 2016-01-08

**Authors:** Alberto Leguina-Ruzzi

**Affiliations:** Hematology and Oncology, Department of Medicine, Pontificia Universidad Católica de Chile, Santiago, Chile, Portugal 61, Second Floor, Santiago, 8330034 Chile

## Abstract

Glutamine is one of the conditionally essential free amino acids with multiple biological functions. Its supplementation to parenteral nutrition has been widely used for the management of complications in intensive care. However, controversial clinical reports have generated reluctance in the use of this pharmaco-nutrient. In this commentary, we address the impact of four studies that influenced the recommendations on glutamine supplementation by the Canadian Clinical Practice Guide 2015. Because of the importance of this guideline in clinical practice, we strongly believe that a more rigorous and critical evaluation is required to support recommendations in future guidelines.

Glutamine (Gln) is a highly abundant free amino acid (AA) with multiple biological functions. It is also one of the conditionally essential AAs, which means that its levels are reduced in the body when under stress or in hypercatabolic conditions [1]. Studies have identified patients in whom a depletion of Gln is correlated with worsening of their pathological condition. Gln depletion is also an independent predictor of mortality in intensive care unit (ICU) patients [2, 3]. Moreover, Rodas et al. [4] showed, in a small observational study, that low (<400 μmol/l) and high (>930 μmol/l) plasma concentrations of Gln in ICU patients are independent risk factors for mortality. It has been reported that high Gln levels are caused by acute liver failure [5] and that in kidney disease the AA clearance is affected, increasing its plasmatic levels [6]. Nevertheless, Gln supplementation for ICU patients with liver or kidney failure (or both) is contraindicated.

Owing to the limited solubility and instability of Gln in aqueous solution [7], it has been supplemented to total parenteral nutrition (TPN) in dipeptide alanylglutamine form. Commonly used TPN products do not provide Gln in their basic composition.

International nutrition guidelines recommend Gln use in ICU patients with TPN requirements. However, controversy has emerged after the publication of the REDOX (Reducing Deaths due to Oxidative Stress) study [8], causing distrust and fear in the use of this supplement.

Recently, a significant change in the guidelines for supplementation of Gln was made by the 2015 Canadian Clinical Practice Guidelines [9]. It is my view that this discouragement of Gln use did not have strong justification.

Quoting the current guidelines, the Critical Care Nutrition panel stated: “when parenteral nutrition is prescribed to critically ill patients, we recommend parenteral supplementation with glutamine NOT be used. There are insufficient data on the use of intravenous glutamine in critically ill patients receiving enteral nutrition but given the safety concerns we also recommend intravenous glutamine not be used in enterally fed critically ill patients” [9].

This change in the 2013 guidelines was influenced by the results of four new trials. The first one, by Carroll et al. [10], is a small study (n = 12 per group) from 2004 that aimed to investigate the effect in anabolism of growth hormone (GH) and insulin-like growth factor (IGF) in the TPN with or without Gln in ICU patients. Interestingly, the authors found that Gln supplementation caused a slight improvement in the net protein balance without any side effect, although no substantial benefit was seen with GH or IGF. Furthermore, they acknowledge the risk implied by the use of these anabolics in TPN but report no evidence of changes in pivotal outcomes such as mortality, ICU length of stay (LOS), or infection.

Secondly, Pérez-Bárcena et al. [11], in a more recent report (2014), showed that suboptimal doses of Gln to TPN for 5 days (with a start delay of more than 28 hours) did not confer any benefit to the ICU patients. However, plasmatic Gln measurement in the patients showed that those with lower levels presented a worse outcome in short-term mortality, LOS, and infections. Moreover, no negative event was reported when Gln was supplemented.

Also in 2014, Grintescu et al. [12] showed that Gln supplementation in trauma patients reduces hyperglycemic episodes and improves insulin response. The authors suggest a role for Gln as an insulin sensitizer and conclude that their results provide evidence for this. Additionally, they highlight some exclusion parameters used in their study (hepatic and kidney dysfunction) that were not considered in the REDOX study.

In the final report which was considered, Koksal et al. [13] in 2014 studied septic patients with a negative nitrogen balance. These patients received enteral nutrition (EN) with or without intravenous (i.v.) Gln and with or without enteral Gln. The authors’ main conclusion was that a double Gln supplementation (i.v. + enteral) on days 7 and 15 improved outcomes for septic patients with malnutrition. Particular improvements were made in transferrin, creatinine, and nitrogen balance. Even when this study mixed TPN and EN with different Gln supplementations, the Gln did not cause any side effects or changes in the clinical outcomes of mortality, infections, or LOS.

From my point of view, four articles that led to changes in the Canadian Nutrition Guidelines did not actually report changes in clinical outcomes relevant to Gln supplementation in TPN. Such outcomes include mortality rates, LOS, and infections. None of the articles reported any negative findings. I believe that even though these well-designed studies give important clinical evidence, a more rigorous and critical evaluation is required to support appropriate decisions in future guidelines.

## Abbreviations

AA: amino acid
EN: enteral nutrition
GH: growth hormone
Gln: glutamine
i.v.: intravenous
ICU: intensive care unit
IGF: insulin-like growth factor
LOS: length of stay
REDOX: Reducing Deaths due to Oxidative Stress
TPN: total parenteral nutrition
