# Erratum to: A commentary on the 2015 Canadian Clinical Practice Guidelines in glutamine supplementation to parenteral nutrition

**DOI:** 10.1186/s13054-016-1203-y

**Published:** 2016-01-25

**Authors:** Alberto Leguina-Ruzzi

**Affiliations:** Hematology and Oncology, Department of Medicine, Pontificia Universidad Católica de Chile, Santiago, Chile, Portugal 61, Second Floor, Santiago, 8330034 Chile

The author would like to issue an erratum for this article [1], and would like to declare the following competing interests which were inadvertently missed in the original publication.

Competing interests

I declare that I worked as an external freelance scientific consultant for Fresenius Kabi in Chile. My work has been limited to bibliographical analysis in the metabolic field non related with clinical practice. I have not endorsed products of Fresenius Kabi company, particularly Dipeptiven.
